# Combined Isolated Laugier's Fracture and Distal Radial Fracture: Management and Literature Review on the Mechanism of Injury

**DOI:** 10.1155/2016/7631425

**Published:** 2016-12-14

**Authors:** Walid Osman, Meriem Braiki, Zeineb Alaya, Nader Naouar, Mohamed Ben Ayeche

**Affiliations:** ^1^Department of Orthopedic Surgery, Sahloul University Hospital, Sousse, Tunisia; ^2^Department of Rheumatology, Farhat Hached University Hospital, Sousse, Tunisia

## Abstract

*Introduction*. Isolated fracture of the trochlea is an uncommon condition requiring a particular mechanism of injury. Its association with a distal radial fracture is rare. We aimed through this case report to identify the injury mechanism and to assess surgical outcomes.* Case Presentation*. We report a 26-year-old female who was admitted to our department for elbow trauma following an accidental fall on her outstretched right hand with her elbow extended and supinated. On examination, the right elbow was swollen with tenderness over the anteromedial aspect of the distal humerus. The elbow range was restricted. Standard radiographs showed an intra-articular half-moon-shaped fragment lying proximal and anterior to the distal humerus. There was a comminuted articular fracture of the distal radius with an anterior displacement. A computed tomography revealed an isolated shear fracture of the trochlea without any associated lesion of the elbow. The patient was surgically managed. Anatomical reduction was achieved and the fracture was fixed with 2 Kirschner wires. The distal radial fracture was treated by open reduction and plate fixation. The postoperative course was uneventful with a good recovery.* Conclusion*. Knowledge of such entity would be useful to indicate the suitable surgical management and eventually to obtain good functional outcomes.

## 1. Introduction

Coronal shear fractures of distal humerus usually involve the capitellum and a variable part of the trochlea [1]. Fracture of the humeral trochlea is usually associated with elbow dislocation or capitellum fracture [2–4]. The trochlea rarely fractures in isolation because of its location deep within the elbow joint and thus is protected from direct trauma [5]. Also called Laugier's fracture, isolated fractures of the articular surface of the trochlea are very uncommon and are sporadically mentioned in the literature as case reports [5–15].

We aimed to document a rare combination of isolated displaced trochlea fracture associated with a distal radial fracture that was successfully managed with open reduction and internal fixation. The literature was discussed and reviewed.

## 2. Case Report

**Figure 1:**
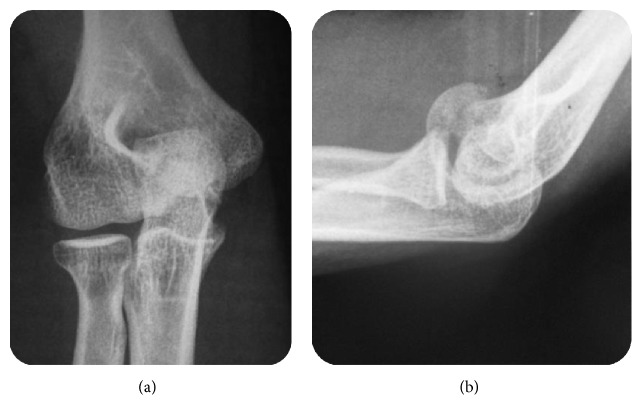
(a) Anteroposterior and (b) lateral radiographs showing an intra-articular half-moon-shaped fragment lying proximal and anterior to the distal humerus simulating a capitellar fracture with irregularities over the trochlear-olecranon articulation surface.

**Figure 2:**
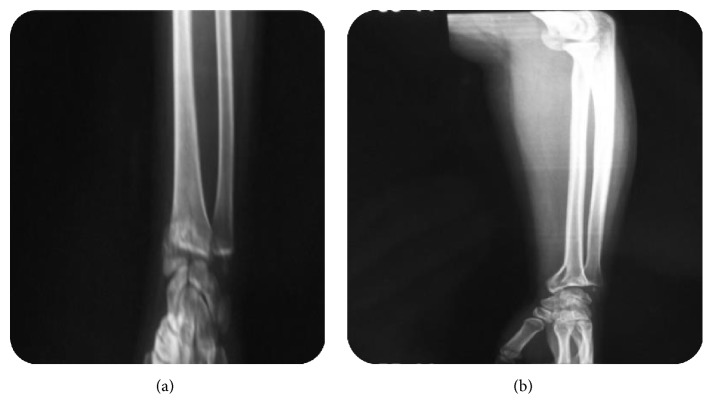
(a) Anteroposterior and (b) lateral radiographs showing comminuted articular fracture of the distal radius with an anterior displacement.

A 26-year-old female was admitted to our department for elbow trauma following an accidental fall on her outstretched right hand with her elbow extended and supinated. She presented with pain and swelling in her right elbow. On examination, the right elbow was swollen with tenderness over the anteromedial aspect of the distal humerus. The elbow range was restricted and too painful. Neurovascular examination was unremarkable. Lateral radiograph (Figure 1) showed an intra-articular half-moon-shaped fragment lying proximal and anterior to the distal humerus simulating a capitellar fracture without associated elbow dislocation. But, on anteroposterior view, the fracture appeared to involve the trochlea showing irregularity of the medial joint space. There was a comminuted articular fracture of the distal radius with an anterior displacement (Figure 2).

**Figure 3:**
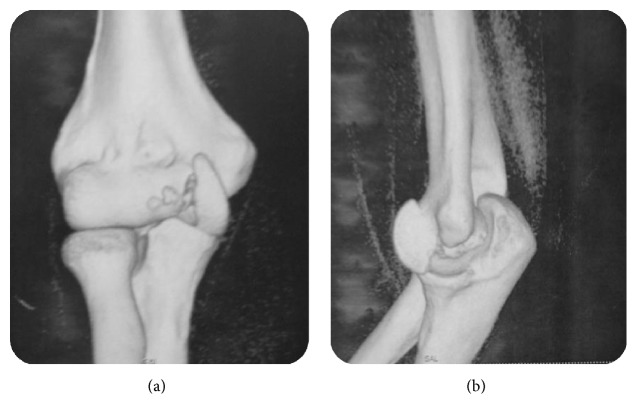
Computed tomography of the elbow with 3D reconstruction showed an isolated shear fracture of the trochlea with no other bony abnormality.

**Figure 4:**
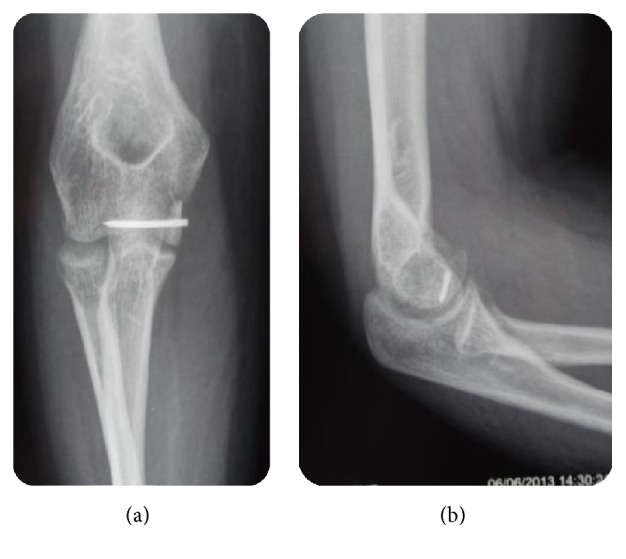
Immediate postoperative radiographs after fixation (a, b) anteroposterior and lateral views of the elbow.

**Figure 5:**
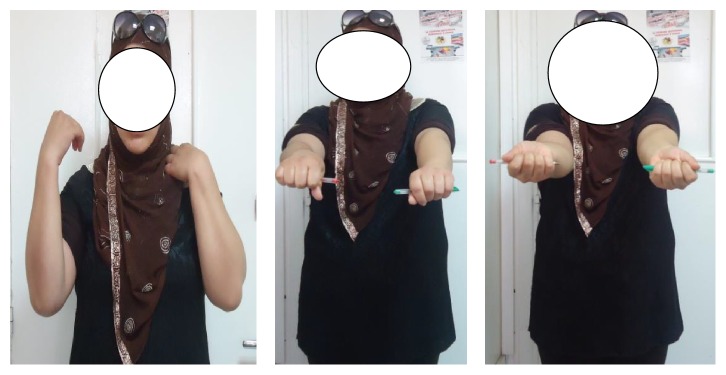
Recovery with excellent functional outcomes.

**Figure 6:**
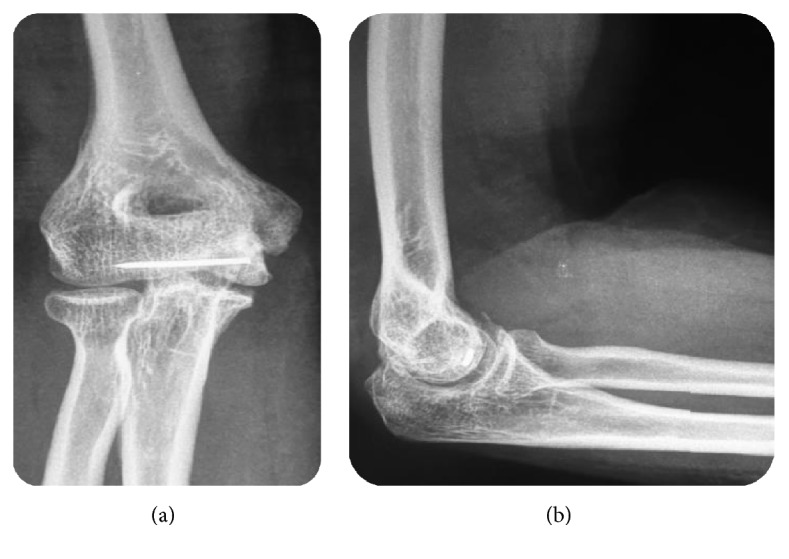
Two years after fixation with Kirschner wires: anteroposterior view (a) and lateral view (b) showing bone union without degenerative changes.

A computed tomography (CT) scan (Figure 3) allowed better analysis of the fracture. It showed an isolated shear fracture of the trochlea without any bony associated lesion of the elbow. The patient was surgically managed. Open reduction and internal fixation were planned for our patient. The fracture site was initially exposed through a medial approach. The ulnar nerve was identified and protected, the common flexors were detached from the medial epicondyle, and the capsule was incised. An osteochondral fragment was displaced proximally. Anatomical reduction was achieved and the fracture was fixed with one Kirschner wire which was directed perpendicular to the fracture line. The reduction of fracture was confirmed preoperatively by direct visualization and fluoroscopic examination (Figure 4). Then, stability and range of movements were checked after the fixation. Common flexors were reattached to the medial epicondyle using nonabsorbable sutures. Subsequently, the distal radial fracture was treated by open reduction and plate fixation through an anterior approach. Postoperatively the limb was immobilized in 90° flexion with an above-elbow back splint for two weeks in order to allow soft tissues healing. Gradual mobilization was started after its removal. The follow-up period was 2 years. At the last control, she was symptoms-free. She turned back to her initial employment. We report excellent functional outcomes according to Dash score which was 83,25/100 (Figure 5). No signs of osteonecrosis or arthritis were found on follow-up radiographs (Figure 6).

## 3. Discussion

Fracture of the trochlea has been previously described as part of the complex fracture of distal end of humerus and dislocation of elbow [1, 7]. However, the trochlea rarely fractures in isolation. This fracture seems to be occurring for several reasons. The trochlea has no muscular or ligamentous attachments and the ulnohumeral joint is not subject to shear forces that occur at the radiocapitellar [5]. Moreover, the trochlea is located deep in the elbow joint; therefore, it is not directly exposed to trauma and usually remains intact in direct elbow injuries [13]. In addition to that, forces transmitted from the ulna across the trochlea tend to produce more a wedging action than shearing forces [8].

Numerous theories were advanced regarding the injury mechanism. Dhurve et al. [15] reported 2 cases of isolated fracture of the trochlea with review of the literature on the mechanism of injury. Authors found three different mechanisms that could be responsible for the occurrence of this fracture: axial load in flexion, axial load in extension, and direct impact of the elbow in flexion. Nakatani et al. [5] suggested the role of varus stress with axial loading in isolated trochlear fracture. Thus, it was documented that varus stress displaces the compressive forces of the radio-humeral compartment in the ulnohumeral compartment [12]. Back to our case, the fracture occurred following an axial load while the elbow was in an extended position.

The mechanisms described in the literature are for isolated fracture of the trochlea. There is only one case report describing a rare combination of an ipsilateral simultaneous fracture of the trochlea involving the lateral end clavicle and distal end radius [7]. In our current case, we think that the possible mechanism might be the preaxial loading stresses in forearm that went to postaxial border of arm and transmitted through elbow which caused fracture of the trochlea with fracture at end distal radius. The patient fell on his hand with his elbow extended; an axial load from the coronoid process and varus stress could have impacted the trochlea.

Clinically, a fracture of the trochlea is atypical with pain, minimal swelling, and tenderness on the medial side of the elbow.

Diagnosis is based on plain radiographs of the injured elbow. It is difficult to detect isolated trochlear fracture on anteroposterior radiograph. However, careful inspection may show an irregularity at the ulnohumeral joint [5, 10, 11] but the image can be interpreted “normal” [6, 8, 13]. A half-moon-shaped osteochondral fragment can be seen on the lateral view which is difficult to distinguish from capitellar fracture. If there is still a doubt, computed tomography (CT) scan can be helpful to make correct diagnosis. In fact, three-dimensional CT reconstructions are very useful for delineating the size and the extent of the fracture more accurately. Moreover, this exam helps rule out other bony injury guiding surgical treatment procedures but cannot show osteochondral damage which is predictive of outcome [3, 6, 9]. In our case report CT scan was useful for surgical planning.

All the cases reported in literature were surgically managed with good outcomes (Table 1). As the osteochondral fracture of the trochlea has no muscular or ligamentous attachments, osteonecrosis is expected in these cases by conventions. However, there is no evidence of osteonecrosis in the reported cases in literature. Conservative treatment has been recommended for undisplaced humeral fracture [4, 11], whereas unsatisfactory results were noted: arthrosis, contracture, and elbow stiffness. If left untreated, the bony fragment can cause mechanical block to elbow flexion by obstructing the coronoid fossa [7]. Excision of irreparable small fragments has been described as a method of treatment. But this may result in a significant loss of articular surface, possibly causing elbow pain and unstability of the ulnohumeral joint [11]. The mainstay of treatment of displaced trochlear fracture is surgical stabilization. Open reduction and stable internal fixation combined with early motion exercises can achieve optimal results [5, 8, 11, 13]. After open reduction, various materials, including Kirschner wires, AO compression screws, and headless compression screws, have been used in the treatment of trochlear fractures with various degrees of success [6]. The choice of materials of fixation depends on the size of the fragment and comminution. Screw fixation allows compression at fracture site with good stability and early motion. Wire fixation is advocated in case of small osteochondral fragment not amenable to screw fixation. After review of the literature, we noticed 15 reported cases, all treated by ORIF. The medial approach was most used in cases. The anterior approach was used only twice. Screw fixation was used in 12 cases and K-wires fixation was used only in 3 cases because of the small size of the fragments. For the same reason, we opted for this type of osteosynthesis and achieved good functional outcomes.

## 4. Conclusion

We treated successfully combined isolated Laugier's fracture and distal radial fracture. A fall on outstretched hand with elbow extended can cause this injury. This case exemplify the excellent functional outcome that is possible with internal fixation and early-range-of-motion exercises.

## Figures and Tables

**Table 1:** Surveys found in the literature reporting details of patients managed for isolated fracture of the trochlea.

Authors	Patients number	Mechanism of injury	Position of elbow during injury	Fixation method	Results
Sen et al. (2013) [6]	5	—	—	Screw fixation and Kirschner wire.	Excellent outcomes in 4 patients and good results in 1 patient.
Gupta et al. (2014) [7]	1	Road traffic accident.	Fall on the outstretched hand. Lying in extending position.	Internal fixation with lag screws.	Good functional outcomes.
Kaushal et al. (2005) [8]	1	Road traffic accident.	Fall on the outstretched hand with the elbow in extension.	Internal fixation with screws.	Excellent functional result according to functional rating scale of Broberg and Morrey.
Somanna et al. (2008) [9]	1	Road traffic accident.	—	Reduction and internal fixation using Kirschner wires.	Excellent functional results.
Foulk et al. (1995) [11]	1	Accidental Fall.	Fall on the outstretched hand with the elbow in extension.	Open reduction with internal fixation.	Good functional results.
Nakatani et al. (2005) [5]	1	Road traffic accident.	Landing on the outstretched hand.	Reduction and internal fixation with Herbert screws.	Good functional results.
Abbassi et al. (2015) [12]	1	Accidental fall.	Landing on the palm of his right hand with his elbow extending and supinated.	Internal fixation.	Good functional results.
Kwan et al. (2007) [13]	2	Road traffic accident.	Landing in a prone position with his elbow in flexed position.	Reduction and internal fixation with Herbert screws.	Good functional results.
Zimmerman et al. (2015) [14]	1	Accidental fall.	Fall on the outstretched hand with the elbow in extension.	Open reduction and internal fixation with two headless Herbert screws.	Good outcomes.

## Additional Points

*Clinical Message*. Isolated Laugier's fractures are uncommon, and their association with distal radial fracture is exceptional. Knowledge about this injury and its mechanism of occurrence is essential to manage adequately this condition. Open reduction and internal fixation are well indicated.
